# Peace impact on health: population access to iodized salt in south Sudan in post-conflict period

**DOI:** 10.3325/cmj.2011.52.178

**Published:** 2011-04

**Authors:** Abdelrahim Mutwakel Gaffar, Mohamed Salih Mahfouz

**Affiliations:** 1 Family and Community Medicine Department, Faculty of Medicine, Jazan University, Jazan, Saudi Arabia; 2 International Council for Control of Iodine Deficiency Disorders, Ottawa, Ontario, Canada; 3 Population Studies Center, Gezira University, Gezira, Sudan

## Abstract

**Aim:**

To determine the population access to salt/iodized salt during and after the armed conflict in south Sudan and to illustrate geographical variations in population consumption of iodized salt in south Sudan after the armed conflict.

**Methods:**

The sources of data for the conflict period were the 2004 Toward a Baseline: Best Estimates of Social Indicators for Southern Sudan study report and the 2000 Multiple Indicators Cluster Survey, and for the post-conflict period the 2005 Sudan Household Health Survey (SHHS) data set.

**Results:**

After peace agreement, population access to salt increased by 6.8% (Z = 5.488, *P* < 0.001) and the consumption of iodized salt increased by 32.9% (Z = 24.668, *P* < 0.001). More than 73% of families were using iodized salt but geographical differences existed between states.

**Conclusion:**

Peace had positive impact on population access to iodized salt in south Sudan. Public health authorities in south Sudan need to establish quality monitoring and surveillance systems to track progress toward Universal Salt Iodization goal defined by the World Health Organization, United Nations Children’s Fund, and the International Council for the Control of Iodine Deficiency Disorders.

War and peace have profound direct and indirect impacts on health (1). In many African countries, civil wars have become endemic; almost 20% of sub-Saharan Africa’s population now lives in countries that are at war (2). Different studies have documented the effect of war on health (3,4).

Sudan is the largest country in Africa with an area of about 2.5 million kilometers. The population of Sudan was 39.2 million in 2008 and the majority (72%) lived in rural areas (5). Before it gained independence in 1956, Sudan had been embroiled in a civil war and after 1956 it was involved in three major civil wars: 1955-72 in Southern Sudan; 1982-2005 in Southern Sudan, Abyei, Nuba Mountains, Blue Nile, and Eastern Sudan; and 2003 in Darfur (6). On 9 January 2005, the Government of Sudan and the Sudan People’s Liberation Movement signed the Comprehensive Peace Agreement, which ended the last civil conflict in the south (7).

It is conceivable that the peace agreement between the Government of Sudan and the Sudan People’s Liberation Movement affected public health in south Sudan. One way to explore this is to look at the changes in population access to iodized salt during armed conflict and in the peace period. Iodine is a natural essential element for human and animal development; it is a constituent of the thyroid hormones, which play a crucial role in growth and development (8). Iodine deficiency is the single greatest cause of preventable mental retardation in the world. Elimination of iodine deficiency contributes to 6 of the 8 Millennium Development Goals defined by the United Nations member states in 2000 (9,10). The national Iodine Deficiency Disorders (IDD) Control Program in Sudan started in October 1989 using Lipiodol in some of the highly endemic regions of the country and adopted salt iodization as the long-term national strategy in 1994 (11). In Sudan, about 1 million newborns yearly (91% of all newborns) are not protected against brain damage. In addition, the goiter prevalence is 22%, which leads to a reduction of up to 25% in the productivity of the affected people (12).

In this study, we used the officially published data by national and international agencies concerning the Universal Salt Iodization program to explore the impact of peace agreement on south Sudan population health using population consumption of iodized salt as a proxy indicator. The specific aims of this study were to determine the changes in the population access/consumption of salt/iodized salt during and after the armed conflict in south Sudan and to illustrate geographical variations in population consumption of iodized salt in south Sudan after the armed conflict.

## Material and methods

This study was based on 3 data sources: the 2005 Sudan Household Health Survey data set (SHHS), the 2004 Toward a Baseline: Best Estimates of Social Indicators for Southern Sudan study report, and the 2000 Multiple Indicators Cluster Survey (MICS) (13-15).

The SHHS is a national survey implemented by the Federal Ministry of Health and the Central Bureau of Statistics, as well as the Ministry of Health together with the Southern Sudan Commission for Census, Statistics and Evaluation. It was the first health survey that covered the entire Sudan after the comprehensive peace agreement. The 2005 SHHS provides information on household, children’s, and women’s health (13). The sample for the SHHS was designed to estimate some key indicators of children’s and women’s health at the national and state level. The sample size in southern states was 9557 households (13).

MICS was a nationally representative survey of households, women, and children. In south Sudan, it covers government control areas and was implemented by the Federal Ministry of Health, Central Bureau of Statistics, and UNICEF in 2000. The objectives of the survey were to assess the position of children and women in Sudan and to furnish the data needed for monitoring progress toward achieving the goals established at the World Summit for Children as a basis for future action. The sample for the 2000 Sudan MICS was designed to estimate a wide range of socio-economic indicators, not only at the national level but also disaggregated by state, urban-rural setting, and sex. Sixteen states in the north and the 3 main towns in the south (Juba, Malakal, and Wau) comprising one unit were covered in the survey, giving a total of 17 geographic units. The sample size of the survey in southern states was 1510 households (14).

Toward a Baseline: Best Estimates of Social Indicators for southern study report was prepared in 2004 by New Sudan Centre for Statistics and Evaluation in association with UNICEF. The report consolidated the data from different sources, including international organizations and local agencies to provide the best estimates of social indicators including health and nutrition status (15).

The data sources for the conflict period were the 2000 MICS and the 1999 MICS for Southern Sudan States, reported in Toward a Baseline: Best Estimates of Social Indicators for Southern Sudan study report (14,15). The 2005 SHHS was used as data source for the post-war period (13).

### Salt testing for iodization and demographic data

The surveys used in this study did not specifically investigate iodine nutrition; we used the demographic data on salt examination, which was only a part in the nutrition section of the surveys. Salt was tested for iodine levels with rapid salt testing kits using color reference indicator in all studies. Rapid salt testing kit is a qualitative method to test the presence of potassium iodate only (10). According to the recent guidelines on assessment of iodine deficiency disorders and monitoring their elimination, the use of a quantitative method for measuring the exact level of iodine in the salt is recommended (10). However, for the purpose of this article we used the officially available data reported in the national reports and the International Network for Sustainable Elimination of Iodine Deficiency (12-17).

### Data analysis

The data were analyzed by the SPSS, version 17 (SPSS Inc., Chicago, IL, USA). Also, we used frequency distribution, as well as Z test to determine if proportions were significantly different from one another. *P* < 0.05 was considered to be significant.

## Results

### Access to salt during conflict and after the 2005 peace agreement

Population access to salt in south Sudan increased significantly, from 64.4% during conflict to 71.2% after the 2005 peace agreement (Z = 5.49, *P* < 0.001). In north Sudan, the access to salt was 97.0% both during and after the conflict. These percentages were significantly higher than in south Sudan for the period during conflict (Z = 57.83, *P* < 0.001) and for the period after the peace agreement (Z = 55.69, *P* < 0.001).

### Iodized salt consumption during conflict and after the 2005 peace agreement

The proportion of households consuming iodized salt in south Sudan increased significantly from 40.0% during conflict to 72.9% after the peace agreement (Z = 24.67, *P* < 0.001). These rates were much lower in north Sudan – 0.6% before and 10.0% after the conflict (Z = 144.17, *P* < 0.001) and in the whole Sudan – 1.0% before and 24.9% after the conflict (Z = 77.92, *P* < 0.001). There were significant differences in iodized salt consumption between south and north Sudan before (Z = 87.39, *P* < 0.001) and after the conflict (Z = 99.84, *P* < 0.001), as well as between south and the whole Sudan (Z = 83.48 before and Z = 82.23 after the conflict, *P* < 0.001 for both periods).

### Geographical variations in the use of iodized salt in south Sudan

According to the 2005 survey, more than 73% of families were using iodized salt, with fewer families using iodized salt in rural areas (67.7%). The proportion of households using iodized salt ranged from 26.11% in Jongolei state to 96.87% in Central Equatoria state (Table 1). Out of 1349 surveyed urban households, iodized salt was used in 1157 (85.8%), and the same was true for 3227 (73.2%) of the 4406 surveyed households in rural areas (Table 1).

**Table 1:** Geographical variations in the number of households using iodized salt in south Sudan in 2005

State	No. (%) of households using iodized salt
Jongolei (n = 360)	94 (26.1)
Upper Nile (n = 103)	90 (87.4)
Unity (n = 362)	211 (58.3)
Warab (n = 273)	211 (77.3)
North Bahr Al Gazal (n = 312)	124 (39.7)
West Bahr Al Gazal (n = 440)	254 (57.7)
Lakes (n = 474)	401 (84.6)
West Equatoria (n = 759)	618 (81.4)
Central Equatoria (n = 957)	927 (96.9)
East Equatoria (n = 366)	297 (81.2)
Total (n = 4406)	3227 (73.2)

### Household sources of salt

About 88% of the surveyed population obtained their salt from the local markets and fewer than 8% from food aid. The proportion of families that obtained their salt from local markets ranged from 75% in Warab state to 99% in West Equatoria state (Table 2).

**Table 2:** The number of households using different sources of salt in geographical areas of south Sudan in 2005

State	No. (%) of households using salt from:			
	local market	food aid	self-production	not known
Jongolei (n = 573)	496 (86.6)	34 (5.9)	19 (3.3)	24 (4.2)
Upper Nile (n = 523)	446 (85.3)	67 (12.8)	2 (0.2)	8 (1.5)
Unity (n = 582)	504 (86.6)	61 (10.5)	0 (0.0)	17 (2.9)
Warab (n = 472)	354 (75.0)	11 (2.3)	1 (0.2)	106 (22.5)
North Bahr Al Gazal (n = 515)	453 (88.0)	34 (6.6)	1 (0.2)	27 (5.2)
West Bahr Al Gazal (n = 641)	512 (79.9)	111 (17.3)	2 (0.3)	16 (2.5)
Lakes (n = 536)	475 (88.6)	51 (9.5)	1 (0.2)	9 (1.7)
West Equatoria (n = 843)	834 (98.9)	6 (0.7)	2 (0.2)	1 (0.1)
Central Equatoria (n = 966)	917 (94.9)	46 (4.8)	0 (0.0)	3 (0.3)
East Equatoria (n = 688)	600 (87.2)	71 (10.3)	1 (0.1)	16 (2.3)
Total (n = 6339)	5591 (88.2)	492 (7.8)	29 (0.5)	227 (3.6)

## Discussion

The study showed changes in the population access to iodized salt as a proxy indicator for the impact of peace on health. These changes illustrate the progress of south Sudan toward Universal Salt Iodization goals.

Population access to salt and consumption of iodized salt have increased significantly after peace agreement in south Sudan, which indicates that population is recovering from war consequences (1,4,18). Access to iodized salt will contribute positively to development efforts in south Sudan by directly increasing the new generation's mental capacities and preventing health, economic, and social consequences of iodine deficiency (8-10,12).

The Universal Salt Iodization strategy to ensure sufficient intake of iodine by all individuals was recommended by the WHO and UNICEF Joint Committee on Health Policy in 1994. Salt iodization is a remarkably cost-effective public health strategy (10). Iodine deficiency increases the risk of cretinism, stillbirth, miscarriage, infant mortality, and brain damage (9). Studies show that the mean IQ score for iodine deficient groups is 13.5 IQ points below that of the non-iodine deficient groups, which is why they will not reach their maximum education potential (8-10). Iodized salt can protect and prevent the population from health, social, and economic consequences of iodine deficiency and hence contribute markedly to the development of communities.

The rapid increase in access to iodized salt after the establishment of peace may be due to the fact that most of the salt in south Sudan is imported from Kenya and Uganda. The proportion of households consuming iodized salt in Kenya was 91% and in Uganda was 96% (17) and the cross-border movement was secured even when the conflict was going on. This fact also explains the geographical variations in the proportion of households using iodized salt in different parts of south Sudan.

Our hypothesis was that most of households depended on food aid agencies, but the study showed that the majority of people (88.2%) obtained salt from local markets, with only 7.8% depending on food aid. This high percentage reflects the growing self-reliance issue in the community.

Using only rapid test kits to determine salt iodine content in all data sources was one of the study limitations. This method needs to be supported by titration method using the standard WHO/UNICEF/ International Council for Control of Iodine Deficiency Disorders guidelines (10). The other limitation was that we could not access MICS 2000 and Multiple Indicators Cluster Survey in south Sudan 1999 data sets to re-analyze it, as we did with SHHS data.

Iodine nutrition survey is urgently needed to explore the situation and identify the quality of salt and iodized salt available on the market. Excessive intake of iodine may cause adverse health consequences (8,10), which can be prevented by quality monitoring and surveillance systems.

In conclusion, peace has had positive impact on population access to salt and iodized salt in south Sudan. The access to iodized salt will positively impact health and economic development of war-affected communities. Public health authorities in south Sudan need to establish quality monitoring and surveillance systems to track progress toward the Universal Salt Iodization goal.
